# 

**Published:** 1893-05

**Authors:** 


### CONTENTS.

ORIGINAL ARTICLES—Asphyxia Neophytorum, an Improved Method of Treatment, by R. J. Nunn, M. D., Savannh, Ga................ 229
Clinic at the Harlem Hospital, by T. H. Manley, M. D., New York 251
Report of Case of Delivery, Complicated by Fibroid Tumor, by T. F. Crofford, M. D., Memphis, Tenn........................ 238
Mercury and the Liver, by A. B. Patterson, M. D., Atlanta, Ga.... 235
SOCIETY REPORTS—Medical Association of Georgia.................. 253
BOOK REVIEWS—Essentials of Nervous Diseases and Insanity, by J. C. Shaw, M. D.............................................. 266
Hand Book of Insanity for Practitioners and Students, by J. Kirchoff, M. D..........................................
Elements of Human Physiology, by E. H. Starling, M. D. . . ....
History of the Life of D. Hayes Agnew, M. D., L. L. D., by J. Howe Adams, M. D., Philadelphia.........................
SELECTIONS AND ABSTRACTS—Mammary Hypertrophy in a Boy........ 265
Gonnorrhoeal Infection of the Mucous Membrane of the Mouth in New-born Infants.......................................... 267
Chloral and Camphor in the Treatment of Chancroid Bromoform in the Treatment of Whoopingcough.......................... 268
EDITORIALS—Yellow Fever Inoculation............................ 269
Medical Association of Georgia.............................. 271
ANNOUNCEMENT.................................................. 275
EDITORIAL NOTES............................................
PRESCRIPTIONS....... ........................................ 277–278
SPECIAL NOTES................................................. 279–280

